# Transforming Growth Factor-β and Long Non-coding RNA in Renal Inflammation and Fibrosis

**DOI:** 10.3389/fphys.2021.684236

**Published:** 2021-05-13

**Authors:** Yue-Yu Gu, Jing-Yun Dou, Xiao-Ru Huang, Xu-Sheng Liu, Hui-Yao Lan

**Affiliations:** ^1^Guangdong Provincial Key Laboratory of Clinical Research on Traditional Chinese Medicine Syndrome, Department of Nephrology, Guangdong Provincial Hospital of Chinese Medicine, Second Affiliated Hospital, Guangzhou University of Chinese Medicine, Guangzhou, China; ^2^Department of Medicine and Therapeutics, Li Ka Shing Institute of Health Sciences, The Chinese University of Hong Kong, Hong Kong, China; ^3^Department of Nephrology, Weihai Hospital of Traditional Chinese Medicine, Weihai, China; ^4^Guangdong-Hong Kong Joint Laboratory for Immunity and Genetics of Chronic Kidney Disease, Guangdong Academy of Medical Sciences, Guangdong Provincial People’s Hospital, Guangzhou, China; ^5^Guangdong-Hong Kong Joint Laboratory for Immunity and Genetics of Chronic Kidney Disease, The Chinese University of Hong Kong, Hong Kong, China

**Keywords:** long non-coding RNA, renal fibrosis, inflammation, TGF-β, SMADs, molecular therapy

## Abstract

Renal fibrosis is one of the most characterized pathological features in chronic kidney disease (CKD). Progressive fibrosis eventually leads to renal failure, leaving dialysis or allograft transplantation the only clinical option for CKD patients. Transforming growth factor-β (TGF-β) is the key mediator in renal fibrosis and is an essential regulator for renal inflammation. Therefore, the general blockade of the pro-fibrotic TGF-β may reduce fibrosis but may risk promoting renal inflammation and other side effects due to the diverse role of TGF-β in kidney diseases. Long non-coding RNAs (lncRNAs) are RNA transcripts with more than 200 nucleotides and have been regarded as promising therapeutic targets for many diseases. This review focuses on the importance of TGF-β and lncRNAs in renal inflammation, fibrogenesis, and the potential applications of TGF-β and lncRNAs as the therapeutic targets and biomarkers in renal fibrosis and CKD are highlighted.

## Introduction

**FIGURE 1:**
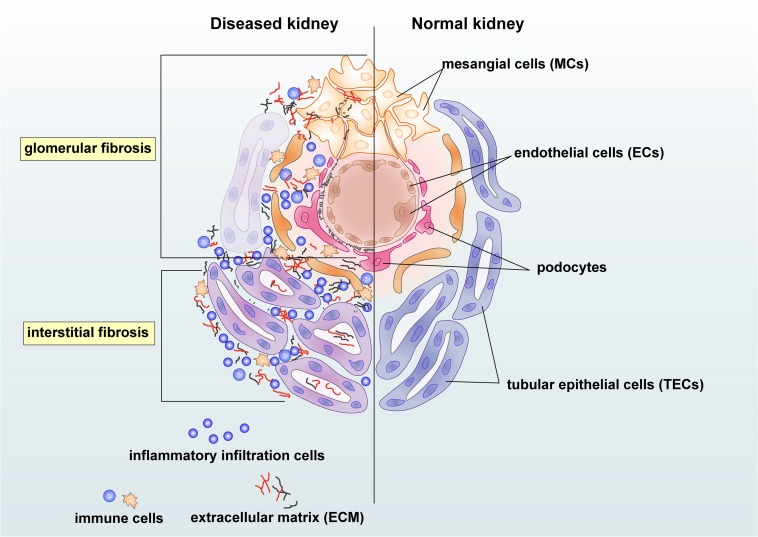
Renal intrinsic and inflammatory cells in glomerular and interstitial fibrosis during kidney injury. Damaged mesangial cells (MCs), endothelial cells (ECs), and podocytes are essential in the glomerular fibrosis. The mesangial cell may produce pro-fibrotic and pro-inflammatory growth factors and cytokines and enhance proliferation to cause deposition of the mesangial matrix. Damages on endothelial cells and podocytes may lead to albuminuria and endothelial dysfunction in chronic kidney disease (CKD). Injured tubular epithelial cells (TECs) may produce pro-fibrotic and pro-inflammatory factors, resulting in the accumulation of extracellular matrix (ECM) and inflammatory cells in the tubulointerstitium area. Immune cells, including macrophages and T cells, may participate in renal fibrosis by producing growth factors and becoming collagen-producing myofibroblasts under the regulation of transforming growth factor-β (TGF-β)/Smad3 signaling.

Chronic kidney disease (CKD) has become a significant public health problem with the rising mortality and morbidity over the past three decades (Provenzano et al., 2019). Renal fibrosis is one of the most prominent pathogenic features and the best predictor for CKD progression (Majo et al., 2019). Triggered by the initial renal insults, the fibrotic process evokes to establish repairs. However, as severe or persistent injuries prolong, renal resident cells, together with infiltrating cells, may contribute to the initiation and progression of fibrosis with excessive deposition of extracellular matrix (ECM) in the glomerulus, tubulointerstitium, and vasculature (Glassock et al., 2017). Moreover, unresolved renal inflammation could also trigger the fibrotic process by releasing pro-fibrotic growth factors, cytokines, and chemokines (Chung and Lan, 2011; Meng et al., 2014). Injuries from mesangial cells, endothelial cells (ECs), podocytes, tubular epithelial cells (TECs), and inflammatory cells could also lead to renal glomerular and interstitial fibrosis (Figure 1). Progressive renal fibrosis and inflammation can then impair the function of nephrons and results in albuminuria and the reduction of eGFR. Renal fibrosis culminates in renal failure, well known as end-stage renal disease (ESRD) (Liu, 2011).

Transforming growth factor-β (TGF-β) is a primary pathophysiologic cytokine that instigates the process of fibrosis (Meng et al., 2016a). TGF-β can induce transcription of fibrotic products such as α-SMA and collagens by canonical and non-canonical signaling pathways. Fibrotic mediators include angiotensin II (Ang II), reactive oxygen species (ROS), as well as advanced glycation end products (AGEs) that may activate individual pathways to crosstalk with TGF-β/Smad signaling to regulate renal fibrosis and inflammation (Chung et al., 2010; Lan, 2011). However, current anti-fibrotic therapies by targeting TGF-β are ineffective with unexpected side effects, underscoring the complexities of the TGF-β signaling pathway (Yoshimura and Muto, 2011; Gu et al., 2020a).

With the new technologies of high-throughput assays, we can now update our understanding of the genomes. The transcriptomic studies have demonstrated that the vast majority of the genomes in mammals produce large numbers of non-protein-coding RNAs (ncRNAs) (Quinn and Chang, 2016). These ncRNAs are classified into long non-coding RNAs (lncRNAs), microRNAs (miRNAs), small interfering RNAs (siRNAs), small nuclear RNAs (snRNAs), small nucleolar RNAs (snoRNAs), and PIWI-interacting RNAs (piRNAs) (Van der Hauwaert et al., 2019). Of these ncRNAs, lncRNAs are characterized as RNAs being transcribed over 200 nucleotides in length. They have been considered the major players in fibrotic diseases’s pathogenesis due to their tissue and cell-type specificity and the regulations on DNAs, RNAs, and proteins (Jiang and Zhang, 2017). Of note, many TGF-β/Smad3-regulated lncRNAs have been reported as essential mediators in the process of renal fibrosis and inflammation (Tang et al., 2017, 2018a, 2018b).

In this review, the underlying mechanistic signaling pathways by which TGF-β and lncRNAs drive renal fibrosis are to be discussed. The developments of biomarkers and therapeutic potential for renal inflammation and fibrosis by targeting TGF-β/Smad signaling and lncRNAs are also described.

## Diverse Roles of TGF-β/Smad Signaling Pathway in Renal Inflammation and Fibrosis

Transforming growth factor-β is a pleiotropic cytokine that plays diverse roles in a wide range of biological and pathological processes. Indeed, TGF-β acts as either deleterious or protective functions in kidney diseases (Lopez-Hernandez and Lopez-Novoa, 2012). TGF-β may induce renal fibrosis by canonical and non-canonical signaling pathways (Lan and Chung, 2012; Isaka, 2018). Besides, TGF-β promotes renal fibrosis by stimulating ECM accumulation and alternatively activating the pro-fibrotic immune cells, facilitating the transitions from various cell types into pro-fibrotic cells (Gu et al., 2020b). Therefore, understanding the diverse roles of TGF-β is of utmost importance in the development of anti-fibrotic therapies.

Transforming growth factor-β is a well-characterized member that belongs to the TGF-β superfamily. Among three isoforms of TGF-β, TGF-β1 is considered the pro-fibrotic molecule that drives the fibrotic process via canonical and non-canonical signaling pathways (Lodyga and Hinz, 2019). In particular, high expression of TGF-β1 is observed in most, not all progressive forms of human and rodent kidney diseases (Kopp et al., 1996; Fan et al., 1999; Lan, 2012b; Lan and Chung, 2012), demonstrating the pathogenic role for TGF-β1 in CKD. To induce transcriptions of target genes, likely as α-SMA and collagens, the latent TGF-β1 becomes active and binds to TGF-β receptors, promoting the transduction of a series of Smad proteins to regulate fibrogenesis (Derynck and Zhang, 2003). Regarding the downstream TGF-β/Smad signaling, although the functions of Smad2 and Smad4 have been well studied (Tsuchida et al., 2003; Ju et al., 2006; Meng et al., 2012; Morishita et al., 2014; Loeffler et al., 2018), their mechanistic roles are diverse and unclear due to the limited availability of animal models, which still warranted for further exploration.

It is widely acknowledged that Smad3 is pro-fibrotic, while Smad2 and Smad7 are anti-fibrotic. Smad3 is highly activated in a wide range of renal disease; evidence on animal models suggest that the inhibition or blockade of Smad3 may reduce the fibrotic response (Wang et al., 2006; Yang et al., 2009, 2010; Li et al., 2010; Zhou et al., 2010; Liu et al., 2012; Zhang et al., 2018). By contrast, the function of Smad2 and Smad7 is protective, which negatively regulates the TGF-β/Smad3 signaling in renal fibrosis and inflammation (Lan, 2008, 2012a; Chen et al., 2011). Many studies support this finding, showing that overexpression of Smad7 improves renal fibrogenesis in obstructive, diabetic, hypertensive, toxin-induced nephropathy and autoimmune crescentic glomerulonephritis (Li et al., 2002; Lan et al., 2003; Hou et al., 2005; Ng et al., 2005; Ka et al., 2007; Chung et al., 2009; Liu et al., 2013, 2014; Dai et al., 2015) by inhibiting the TGF-β/Smad3 and NF-κB signaling pathways. However, the contradictory findings have also reported that the overexpression of Smad7 could promote TGF-β-driven apoptosis in podocytes (Schiffer et al., 2001, 2002). Collectively, although restoring the imbalance between Smad3 and Smad7 may serve as an ideal therapy to halt the fibrotic process (Nie et al., 2014; Zhao et al., 2014, 2016; Meng et al., 2015; Du et al., 2018b), Smad3 and Smad7 also serve as the vital downstream molecules in other signaling pathways. Therefore, new specific targets should be sought.

Transforming growth factor-β may also be produced by damaged renal intrinsic cells or immune cells in acute and CKDs, thus promoting the transition of tubular cells into myofibroblasts (Mack and Yanagita, 2015). Myofibroblasts produce fibronectin and collagens and contribute to ECM accumulation (Yuan et al., 2019). Based on current studies, the sources of myofibroblast origins include pericytes (Wu et al., 2013), renal resident fibroblasts, tubular epithelial cell-myofibroblast transition (EMT) (Iwano et al., 2002), endothelial cell-myofibroblast transition (EndoMT) (Zeisberg et al., 2008) and bone marrow-derived macrophage-myofibroblast transition (MMT) (Fan et al., 1999; Meng et al., 2016b; Wang et al., 2017). TGF-β/Smad signaling pathway tightly regulates these transitions.

To halt the fibrotic process, strategies to inhibit the function of TGF-β include the utilization of neutralizing antibodies (Border et al., 1990), small molecule inhibitors against TGF-β receptors (Bonafoux and Lee, 2009), latent form of TGF-β (Huang et al., 2008a, b) and antisense oligonucleotides to TGF-β1 (March et al., 2018). These findings have conferred a vital pathological role of TGF-β in renal inflammation and fibrosis, implying the urgent need for anti-TGF-β therapy.

## Therapeutic Effect of Anti-TGF-β Treatment on Kidney Diseases

Anti-TGF-β therapy is an issue of considerable debate. On the one hand, TGF-β is the crucial mediator that regulates fibrosis in all organs, especially in kidneys (Györfi et al., 2018). On the other hand, TGF-β regulates a wide range of biological and pathological processes and acts as essential roles in the immune cells, such as macrophages, conventional and unconventional T cells (Meng, 2019; Gu et al., 2020a). Over the past decades, a number of therapeutic drugs and clinical trials for the treatment of CKD targeting TGF-β have further revealed the underlying mechanisms and renewed our understanding of TGF-β signaling (Ruiz-Ortega et al., 2020).

Targeting on the TGF-β family, LY2382770 and fresolimumab have proven no efficacy on improvements in neither proteinuria, eGFR, nor serum creatinine in focal and segmental glomerulosclerosis (FSGS) and diabetic nephropathy (DN) (Trachtman et al., 2011; Vincenti et al., 2017; Voelker et al., 2017). Besides, various side effects induced by blocking TGF-β, including herpes zoster, skin lesions, pustular rash, bleeding events, and cancers, have demonstrated the awkward situation of the anti-TGF-β therapies. Hopefully, with the rapid development of pharmacology, a promising synthetic anti-TGF-β agent, pirfenidone, is proven to improve the eGFR decline in patients with DN and FSGS (Cho et al., 2007; Sharma et al., 2011). Further studies and clinical trials on pirfenidone’s renal protective effects are still ongoing (NCT02689778, NCT02408744, and NCT00001959).

Nevertheless, the by-effects such as gastrointestinal disorders and photosensitive dermatitis of pirfenidone are inevitable, raising safety concerns to the clinical application of anti-TGF-β therapies. Current anti-TGF-β therapies have limited effectiveness, underscoring the urgent need to develop specific therapeutic targets to halt the progression of renal fibrosis.

## The Emerging Role of Long Non-Coding RNAs in Renal Inflammation and Fibrosis

The genomic and transcriptional landscape is far more complicated than we previously appreciated. With the development of large-scale transcriptome analyses, we have now acknowledged that the vast majority of genomic sequence is transcribed into a group of lncRNAs (Hangauer et al., 2013). However, these lncRNAs were initially ignored as “transcriptional noise” or “evolutionary debris,” dating from the 1970s (Ohno, 1972). In the 1990s, the functions of some classically defined lncRNAs are discovered, such as X inactive specific transcript (XIST) in X chromosome inactive specific, raising the possibility that lncRNAs may play an essential role in cellular biology and disease (Brockdorff et al., 1991; Brown et al., 1991). Of note, the number of identified lncRNAs is rapidly rising to date. Based on the GENCODE^1^ (version 33), 17952 lncRNA and 19957 protein-coding genes have been identified in the human genome, but the functions of lncRNAs in renal development and diseases remain largely unknown. In the context of lncRNA function in kidney diseases, lncRNAs may act as scaffolds, decoys, or guides to control the recruitment or dismissal of chromatin-modifying complexes.

Although lncRNAs produce in deficient amounts, their expression patterns are highly restricted to specific cell types, tissue, developmental stage, or disease state, suggesting the distinctive roles of lncRNAs in different physiological or pathological contexts (Batista and Chang, 2013; Flynn and Chang, 2014). Pathologically, the fibrotic and inflammatory processes in the kidneys may be triggered by a wide range of renal injuries in the attempt to establish tissue repair. Pathological hallmarks include TGF-β activation, myofibroblast differentiation and transition, ECM deposition, and inflammatory responses. Of note, TGF-β is a master regulator of immune cell trades that it correlates closely with the development, homeostasis, and differentiation of immune cells such as T cells (Li and Flavell, 2008). T cells are the predominant players in TGF-β-driven renal fibrosis and inflammation (Kinsey and Okusa, 2014; Ludwig-Portugall and Kurts, 2014; Hu et al., 2016). The hematopoietic-specific TGF-β and cytokines produced by inflammatory immune cells may activate innate and acquired immune response (Gu et al., 2020b). These may well be associated with the functions of lncRNAs in renal inflammation. For instance, lncRNAs may act as mediators in lupus nephritis pathogenesis to regulate inflammation and apoptosis of renal cells (Xue et al., 2017; Liao et al., 2019; Chen et al., 2020).

Nevertheless, lncRNAs take part in the fibrotic or inflammatory transcriptional regulation by direct interactions with RNA polymerase II (Pol II), transcription factors (TFs), and other regulators. Furthermore, some lncRNAs may act as competing endogenous RNAs (ceRNAs), which play the competitive role as the sponges to bind with miRNAs and reduce the concentration of fibrotic or inflammatory miRNAs, therefore competing with these miRNAs in binding to their target mRNA transcripts.

As previously mentioned, the group of lncRNAs identified in kidneys is highly specific to cell type or disease state. Studies carried out over these years have identified a group of anti- or pro-fibrotic and inflammatory lncRNAs in diabetic, acute and chronic renal diseases (Tang et al., 2017, 2018a; Ren et al., 2019; Gu et al., 2020c) (Table 1).

**TABLE 1:** Anti-fibrotic or anti-inflammatory long non-coding RNAs (lncRNAs) in renal diseases.

**lncRNA**	**Model**	**Mechanism/target**	**Pathological output(s)**	**Year**	**References**
GAS5	STZ-induced DN and rat	Recruits EZH2 to the promoter region of MMP9	Anti-fibrotic; anti-inflammatory	2020	Zhang et al., 2020a
CRNDE	Sepsis-induced AKI, rat, and TECs	Regulation of miR-181a-5p/PPARα pathway	Anti-inflammatory	2020	Wang et al., 2020a
CCAT1	LPS-induced AKI mice and TECs	Overexpression of CCAT1 sequesters miR-155 and leads to upregulation of SIRT1 and TECs damage	Anti-inflammatory	2020	Lu et al., 2020
TUG1	SLE patient serum and SLE mouse	/	Anti-fibrotic; anti-inflammatory	2020	Cao et al., 2020a, b
	LPS-induced podocyte injury	Targets miR-197/MAPK1	Anti-inflammatory	2019	Zhao et al., 2019
Rian/RIAN	UUO mouse, AKI mouse, and pericytes	Possible interactions with 14q32 miRNA cluster	Anti-fibrotic	2019	Bijkerk et al., 2019
Malat1/MALAT1	AKI; mice; and TECs	Regulates HIF-1α expression through NF-κB signaling	Anti-inflammatory	2018	Tian et al., 2018
ZEB1-AS1	DN mouse and DN patient	Binds to H3K4 methyltransferase myeloid and MLL1 to promote ZEB1 expression Provides a binding site for p53	Anti-fibrotic	2018	Wang et al., 2018a
1700020I14Rik	DN mouse and MCs	Interacts with miR-34a-5p, Sirt1/HIF-1α	Anti-fibrotic	2018	Li et al., 2018
CYP4B1-PS1-001	DN mouse and MCs	Regulates Nucleolin to inhibit proliferation and fibrosis of MCs	Anti-fibrotic	2018 2016	Wang et al., 2016a, 2018c
3110045C21Rik	UUO mouse and TECs	Contains binding sites for Pol II and H3K4m3	Anti-fibrotic	2016	Arvaniti et al., 2016
ENSMUST00000147869	DN mouse and MCs	Possibly targets on *Cyp4a12a* gene	Anti-fibrotic	2016	Wang et al., 2016b

For example, hyperglycemia is one of the most driving forces in renal fibrosis. Zhang et al. has revealed the anti-fibrotic effect of lncRNA growth arrest-specific transcript (GAS5) in the progression of DN. lncRNA GAS5 may downregulate the expression of pro-inflammatory MMP9 by recruiting EZH2 to the MMP9 promoter region, therefore inhibiting renal interstitial fibrosis and inflammatory (Zhang et al., 2020a). lncRNA CRNDE also interacts with miR-181a-5p to to protect sepsis-induced AKI from apoptosis (Wang et al., 2020a). Moreover, overexpression of lncRNA CCAT1 may down-regulate miR-155, thus inhibiting inflammation and promoting proliferation (Lu et al., 2020). LncRNA zinc finger E-box binding homeobox1-antisense RNA 1 (ZEB1-AS1) provides a binding site in its promoter region for p53. It may promote H3K4me3 histone modification on ZEB1 promoter to exhibit anti-fibrotic effect (Wang et al., 2018a). In the context of fibrosis, the function of lncRNA metastasis-associated lung adenocarcinoma transcript 1 (MALAT1) has been well-studied in cardiac and in hepatic fibrosis (Jiang et al., 2019; Riaz and Li, 2019; Che et al., 2020). MALAT1 has caught much attention in renal diseases for its anti-inflammatory effect in AKI. The expression of LncRNA 1700020I14Rik tends to decrease under high glucose conditions, but the overexpression of LncRNA 1700020I14Rik exerts an anti-fibrotic effects by inhibiting cell proliferation and regulating the miR-34a-5p/Sirt1/HIF-1α pathway (Li et al., 2018). Moreover, lncRNA CYP4B1-PS1-001 significantly reduces in the early stage of DN; the proliferation and fibrosis of mesangial cells are reversed as the overexpression of CYP4B1-PS1-001 regulates the ubiquitination and degradation of Nucleolin (Wang et al., 2016a, 2018c). ENSMUST00000147869 is significantly downregulated in the DN model. Overexpression of ENSMUST00000147869 may inhibit fibrosis and proliferation of mesangial cells by the possible regulation of the Cyp4a12a gene (Wang et al., 2016b). To interact with miRNAs and proteins in podocytes, pericytes, or TECs, lncRNAs such as taurine upregulated gene 1 (TUG1) (Zhao et al., 2019; Cao et al., 2020a, b), Rian (Bijkerk et al., 2019), 3110045C21Rik (Arvaniti et al., 2016) also function as anti-fibrotic lncRNA to participate in the pathogenesis of systematic erythematosus lupus (SLE), ischemia-reperfusion injury and obstructive nephropathy, respectively.

Studies on pro-fibrotic lncRNAs are shown in Table 2. LncRNA myocardial infarction-associated transcript (Miat) has been identified to function as miRNA sponges in TECs and pericytes, thus regulating their transitions into myofibroblast (Bijkerk et al., 2019; Wang et al., 2020b). In diabetes-induced renal injury, lncRNA nuclear enriched abundant transcript 1 (NEAT1) is found to be increased in the serum of DN patients. A further mechanistic study has revealed that lncRNA NEAT1 may progress the development of DN by sponging miR-23c. Moreover, studies from Yang et al. and Huang et al. have drawn similar conclusions. At the same time, they further demonstrated that lncRNA NEAT1 might promote fibrosis in TECs and MCs by regulating the ERK1/2 or Akt/mTOR signaling pathways (Huang et al., 2019; Yang et al., 2020). It is reported that NEAT1 may also promote renal inflammation in lupus nephritis by upregulating the expression of TRAF6 and activating the NF-κB signaling in lupus nephritis (Zhang et al., 2020b). lncRNA LOC105375913 is upregulated in the TECs of FSGS patients and functions to promote tubulointerstitial fibrosis by regulating C3a/p38/XBP signaling pathway and by increasing the expression of Snail and binding to miR-27b (Han et al., 2019). lncRNA LINC00667 is also upregulated in kidney tissues related to the proliferation of TECs. A further mechanistic study has revealed that LINC00667 promotes renal fibrosis by regulating the miR-19b-3p/LINC00667/CTGF signaling pathway (Chen et al., 2019). lncRNA TapSAKI is reported as a biomarker with pro-inflammation and pro-apoptosis in injured TECs and can predict mortality in AKI patients (Lorenzen et al., 2015a).

**TABLE 2:** Pro-fibrotic or pro-inflammatory lncRNAs in renal diseases.

**lncRNA**	**Model**	**Mechanism/function**	**Pathological output(s)**	**Year**	**References**
Miat/MIAT	UUO mouse and TECs	Sponge for miR-145	Pro-fibrotic	2020	Wang et al., 2020b
	UUO mouse, IRI mouse, and pericytes	Possible interactions with miR-150	Pro-fibrotic	2019	Bijkerk et al., 2019
Neat1/NEAT1	DN mouse and TECs	Regulates the Klotho/ERK1/2 signaling	Pro-fibrotic	2020	Yang et al., 2020
	Plasma from DN patient, DN mouse, and MCs	Sponge for miR-23c	Pro-fibrotic	2020	Li et al., 2020
	DN rat and MCs	Possible regulation of Akt/mTOR	Pro-fibrotic	2019	Huang et al., 2019
	MCs	Targets miR-146b to promote TRAF6 expression	Pro-inflammatory	2019	Zhang et al., 2020b
LOC105375913	FSGS patient and TECs	Regulated by C3a/p38/XBP-1s signaling and binds to miR-27b	Pro-fibrotic	2019	Han et al., 2019
LINC00667	CKD patient, CKD rat, and TECs	Promotes fibrosis via miR-19b-3p/LINC00667/CTGF signaling	Pro-fibrotic	2019	Chen et al., 2019
TapSAKI	Sepsis-induced AKI; rats; and TECs	Promotes apoptosis and inflammation of TECs via TaqSAKI/miR-22/TLR4/NF-κB signaling pathway	Pro-inflammatory	2019	Shen et al., 2019
Rpph1	db/db mice and MCs	Promotes inflammation and MCs proliferation through Gal-3/Mek/Erk signaling	Pro-inflammatory	2019	Zhang et al., 2019b
Blnc1	DN patient, STZ-induced DN, and TECs	Interaction with NRF2/HO-1 and NF-κB signaling	Pro-fibrotic; Pro-inflammatory	2019	Feng et al., 2019
Malat1/MALAT1	DN and TECs	Regulation of Wnt/β-catenin signaling	Pro-fibrotic	2019	Zhang et al., 2019a
	DN mouse and TECs	Sponge for miR-145	Pro-fibrotic	2019	Liu et al., 2019a
	Plasma, renal biopsies from AKI patients, IRI mouse, TECs, and ECs	Regulated by HIF-1α	No significant effect	2018	Kölling et al., 2018
	DN mouse and podocytes	Binds to SRSF1, interacts with β-catenin	Pro-fibrotic	2017	Hu et al., 2017
	STZ-induced mice and ECs	Upregulated IL-6, TNF-α by activating SAA3	Pro-inflammatory	2015	Puthanveetil et al., 2015
ENSRNOG00000037522	DN rat and podocytes	/	Pro-fibrotic	2018	Ling et al., 2018
NR_033515	Serum from DN patient and MCs	Negatively regulates miR-743b-5p	Pro-fibrotic	2018	Gao et al., 2018
LINC00963	5/6 nephrectomy and rat	Activates the FoxO signaling	Pro-fibrotic	2018	Chen et al., 2018
CHCHD4P4	Kidney stone, mouse and TECs	/	Pro-fibrotic	2017	Zhang et al., 2017a
ASncmtRNA-2	DN mouse and MCs	Upregulated by ROS	Pro-fibrotic	2017	Gao et al., 2017
Gm4419	DN mouse and MCs	Activates NF-κB/NLRP3-mediated inflammation and interacts with p50	Pro-fibrotic; pro-inflammatory	2017	Yi et al., 2017
PVT1	AKI; and LPS-induced TECs	Binds to TNF-α and inhibits JNK/NF-κB signaling pathway	Pro-inflammatory	2017	Huang et al., 2017
RP23-45G16.5	UUO mouse and TECs	Shows positive correlation with *cdkn1b* gene	Pro-fibrotic	2016	Arvaniti et al., 2016

In diabetic kidney disease, ribonuclease P RNA component H1 (Rpph1) (Zhang et al., 2019b)and Blnc1 are marked as pro-inflammatory lncNRAs (Feng et al., 2019). Moreover, studies on DN have observed the upregulation of lncRNA MALAT1 in TECs and podocytes under high glucose-induced conditions. Induced by TGF-β1, MALAT1 facilitates EMT and promotes fibrosis by acting as a sponge for miR-145 or as a feedback regulator of the Wnt/β-catenin signaling pathway (Hu et al., 2017; Liu et al., 2019a; Zhang et al., 2019a). However, the pathogenic role of lncRNA MALAT1 in hypoxia-induced AKI remains unclear. Kölling et al. have identified an increased level of lncRNA MALAT1 in renal biopsies and plasma of AKI patients; *in vitro* study has also shown a decreased number and proliferation in MALAT1-inhibited ECs. The mechanistic study has discovered that it is transcriptionally activated by hypoxia-inducible factor 1α (HIF-1α). However, no significant differences in inflammation and fibrosis were shown on MALAT1 knockout and wild-type mice in hypoxia-induced AKI (Kölling et al., 2018).

Also, microarray data have shown a pro-fibrotic role of lncRNA LINC00963 by targeting on FoxO3 gene to regulate the FoxO signaling pathway (Chen et al., 2018). Pro-inflammatory cytokines, together with NLRP3 inflammasome, may also drive the progression of fibrosis under diabetic conditions. Furthermore, lncRNA Gm4419 is increased in DN and promotes renal fibrosis and inflammation by activating the NF-κB/NLRP3 inflammasome signaling pathway in MCs (Yi et al., 2017). However, the functional roles of lncRNA ENSRNOG00000037522 and CHCHD4P4 are remained to be further investigated (Zhang et al., 2017a; Ling et al., 2018).

## Transforming Growth Factor-β/Smad3-Dependent LncRNA in Renal Inflammation and Fibrosis

Fibrotic responses triggered by TGF-β/Smad3 signaling are of importance in renal fibrogenesis. However, generally blocking the upstream TGF-β signaling may risk promoting inflammation and other side effects. We are beginning to learn that the involvement of TGF-β in many other biological processes has been the main obstacle for anti-TGF-β therapy. Nevertheless, the majority of studies continue to seek therapeutic targets for anti-fibrotic treatments. miRNA targeting downstream TGF-β signaling has been one of the optimal options.

However, the off-target effects and cytotoxicity of miRNA therapies have caught the attention of their specificity and safety. Encouragingly, it has been reported that a group of characterized lncRNAs is involved in TGF-β/Smad3-mediated renal fibrosis and inflammation (Zhou et al., 2014, 2015b) (Table 3). These emerging studies should provide possibilities for lncRNA treatment in the future. Ptprd-IR is a novel lncRNA that promotes inflammatory response on TECs in the UUO model. It contains a binding site for Smad3 in its promoter region and is downregulated by deleting Smad3. In contrast, the overexpression of Ptprd-IR enhances inflammatory response by upregulating TGF-β1-, interleukin-1β (IL-1β)-induced NF-κB-driven production of pro-inflammatory cytokines but shows no effect on the TGF-β1-induced renal fibrosis (Pu et al., 2020). Other novel lncRNA, lncRNA Erbb4-IR, of which expression is induced by TGF-β1 via Smad3-dependent mechanism, is significantly increased in the fibrotic UUO model (Feng et al., 2018). Erbb4-IR binds to the inhibitory Smad7 and blocks TGF-β/Smad3-induced renal fibrosis, while overexpression of Erbb4-IR may promote fibrosis by downregulating the expression of Smad7. Of note, Erbb4-IR may also be induced by advanced glycosylation end products (AGEs) in DN. It promotes the expression of collagens by binding to miR-29b and hence transcriptionally suppresses miR-29b. Silencing renal Erbb4-IR leads to the upregulation of protective miR-29b and prevents fibrosis (Sun et al., 2018; Xu et al., 2020). Besides, lncRNA AT-rich interactive domain 2-IR (Arid2-IR) also contains a Smad3 binding site in the promoter region. Further *in vivo* study has shown that deletion of Smad3 may abolish upregulation of Arid2-IR in the diseased kidney. Arid2-IR shares a similar mechanism with Ptprd-IR that overexpression of Arid2-IR may promote TGF-β1-, IL-1β-induced NF-κB-driven inflammation without affecting TGF-β/Smad3-mediated renal fibrosis (Zhou et al., 2015a). Nevertheless, the study from Yang et al. (2019) has demonstrated the upregulation and pro-fibrotic effect of Arid2-IR on MCs in DN, that Arid2-IR may be positively regulated by the early growth response protein-1 (Egr1) and promote ECM production.

**TABLE 3:** TGF-β/Smad3-dependent lncRNAs in renal fibrosis and inflammation.

**ncRNA**	**Model**	**Mechanism/function**	**Pathological output(s)**	**Year**	**References**
Ptprd-IR (np_4334)	UUO mouse and TECs	Contains a binding site for Smad3 and promotes NF-κB-driven inflammation	Pro-inflammatory	2020	Pu et al., 2020
Erbb4-IR (np_5318)	DN mouse, TECs, and MCs	Binds to miR-29b to downregulate miR-29b expression	Pro-fibrotic	2020 2018	Sun et al., 2018; Xu et al., 2020
	UUO mouse and TECs	Binds to Smad7 to downregulate Smad7 expression	Pro-fibrotic	2018	Feng et al., 2018
Arid2-IR (np_28496)	DN mouse and MCs	Upregulated by Egr-1-induced ECM production	Pro-fibrotic	2019	Yang et al., 2019
	UUO mouse, anti-GBM mouse, and TECs	Contains a binding site for Smad3 and promotes NF-κB-driven inflammation	Pro-inflammatory	2015	Zhou et al., 2015a
LRNA9884	DN mouse, TECs, and MCs	Directly triggers the MCP-1 production	Pro-inflammatory	2019	Zhang et al., 2019c
NONHSAG053901	DN mouse and MCs	Directly binds to Egr-1	Pro-fibrotic; pro-inflammatory	2019	Peng et al., 2019
HOTAIR	UUO rat and TECs	Regulation of miR-124/Notch1	Pro-fibrotic	2019	Zhou et al., 2019
lncRNA-ATB	UUO rat and TECs	Regulated by Livin to promote EMT	Pro-fibrotic	2019	Zhou and Jiang, 2019
MEG3	TECs	Regulated by miR-185/DNMT1 axis to inhibit fibrosis	Anti-fibrotic	2019	Xue et al., 2019
	TECs; acute renal allograft; and mice	Function as target of miR-181b-5p to regulate the expression of TNF-α	Pro-inflammatory	2019	Pang et al., 2019
ENST00000453774.1	Human renal fibrotic tissue, UUO mouse, and TECs	Activates autophagy by promoting ROS defense activates Nrf2/HO-1 signaling	Anti-fibrotic	2019	Xiao et al., 2019
lnc-TSI	IgAN patient and UUO mouse	Binds with Smad3 to block the interaction between Smad3 and TβR1	Anti-fibrotic	2018	Wang et al., 2018b
TCONS_00088786	UUO mouse and TECs	Possible regulation of miR-132	Pro-fibrotic	2018	Zhou et al., 2018
	UUO rat and TECs	/	Pro-fibrotic	2017	Sun et al., 2017
TCONS_01496394	UUO rat and TECs	/	Pro-fibrotic	2017	Sun et al., 2017
H19	UUO mouse, DN mouse, and TECs	Stimulated by TGF-β2 and serves as a sponge for miR-17	Pro-fibrotic	2016	Xie et al., 2016

*CKD, chronic kidney disease; FSGS, focal and segmental glomerulosclerosis; STZ, streptozotocin; DN, diabetic nephropathy; SLE, systematic erythematosus lupus; IgAN, IgA nephropathy; anti-GBM, anti-glomerular basement membrane; LPS, lipopolysaccharides; UUO, unilateral ureteral obstruction; AKI, acute kidney injury; IRI, ischemia-reperfusion injury; MCs, mesangial cells; TECs, tubular epithelial cells; ECs, endothelial cells; SAA3, serum amyloid antigen 3.*

A novel Smad3-dependent lncRNA, LRNA9884, is induced by AGEs and tightly regulated by Smad3 in the development and progression of DN. Mechanistically, LRNA9884 directly binds to MCP-1 and enhances the promoter activity of MCP-1 at the transcriptional level, thus aggravating the renal injury driven by progressive inflammation (Zhang et al., 2019c). The kidney-enriched TGF-β/Smad3-interacting lncRNA, term as Inc-TSI, is another novel lncRNA that serves as a potential target for renal fibrosis (Wang et al., 2018b). lnc-TSI inhibits renal fibrosis by binding to the MH2 domain of Smad3, therefore blocking the interaction of Smad3 and TβRI and inhibiting the phosphorylation of Smad3. Meanwhile, the overexpression of lnc-TSI prevents the nuclear translocation of Smad2/3/4, resulting in the decreased expression of fibrotic proteins. The anti-fibrotic role of lnc-TSI has further confirmed that the fibrosis index of IgAN patients is negatively correlated with the expression of lnc-TSI.

Collectively, the TGF-β/Smad3-mediated lncRNAs may act as anti-fibrotic or pro-fibrotic mediators in the fibrotic process by binding to Smad3, Smad7, or inflammatory molecules to inhibit or enhance renal fibrosis and inflammation. It has been demonstrated by a large number of studies that lncRNAs act like an endogenous RNA to compete for miRNA to regulate the target transcripts at the transcriptional or post-transcriptional level during renal fibrosis. In the early stage of DN, the expression of lncRNA NONHSAG053901 is highly increased in DN mice and MCs. The functional study has revealed that the overexpression of NONHSAG053901 promotes fibrosis, inflammation, and proliferation in MCs. Mechanistically, NONHSAG053901 directly binds to Egr-1, which later interacts with TGF-β to upregulate the release of pro-inflammatory cytokines to promote Egr-1/TGF-β mediated renal inflammation (Peng et al., 2019). In addition, the pro-fibrotic lncRNA HOTAIR is significantly upregulated in TGF-β1-induced TECs and UUO rat kidney. Depletion on HOTAIR upregulates miR-124 to block the Notch1 signal pathway, therefore improving the EMT and reducing the accumulation of fibrotic proteins such as fibronectin and α-SMA (Zhou et al., 2019).

lnc-ATB has also been proven to be the critical regulator stimulated by TGF-β that mediates the EMT process. The expression of lncRNA-ATB is significantly increased in TECs and the UUO kidney under TGF-β and Livin regulation (Zhou and Jiang, 2019). Another lncRNA regulated by TGF-β is maternally expressed gene 3 (MEG3), inhibited in TGF-β-stimulated TECs. DNA methyltransferases 1 (DNMT1), regulated by miR-185, can positively modulate the methylation state of CpG islands in the promoter region of MEG3. Overexpression of lncRNA MEG3 reverses TGF-β-induced fibrosis in TECs. Thus, lncRNA MEG3 exerts an anti-fibrotic effect in TGF-β-promoted EMT and is regulated by the miR-185/DNMT1 signaling pathway (Xue et al., 2019). However, one study had investigated the pro-inflammatory effect of MEG3 in the acute renal allograft model (Pang et al., 2019). The anti-fibrotic lncRNA, ENST00000453774.1, is also downregulated in TGF-β-induced TECs and UUO model, especially in the fibrotic renal biopsies from patients. ENST00000453774.1 may regulate the Nrf2-keap1/Nrf2 nuclei translocation/HO-1 and NQO-1 signaling to activate the pro-survival autophagy of TECs, therefore promoting ROS defense and reducing the production of ECM markers such as fibronectin and collagen I (Xiao et al., 2019).

Nevertheless, the mechanism of some pro-fibrotic lncRNAs is still obscure. Based on the transcriptome sequencing study, a group of lncRNAs that contain Smad3 binding motifs in the promoter region has been identified. Among these lncRNAs, TCONS_00088786 and TCONS_01496394 are confirmed to be regulated by TGF-β in a time and dose-dependent manner. Knockdown of TCONS_00088786 may inhibit the mRNA expression profile of the gene Acta1, Col1a1, and Col3a1, while knockdown of TCONS_01496394 decreases the mRNA expression of Ctgf and Fn1, suggesting their potential in promoting renal fibrosis (Sun et al., 2017). Although a functional study has shown a positive regulation of TCONS_00088786 on miR-132, the underlying mechanism is unclear (Zhou et al., 2018). Interestingly, the expression of lncRNA H19 is also increased in TECs and the UUO model. lncRNA H19 is activated in embryonic cells, but its expression is significantly decreased after birth. Under the renal fibrotic condition, H19 is upregulated by TGF-β2 to promote the production of ECM-related proteins. Knockdown of H19 restores the renal functions and inhibits TGF-β2-induced fibrosis. It is demonstrated that H19 serves as a sponge for miR-17 and negatively regulates miR-17 in the process of fibrogenesis (Xie et al., 2016). However, further evidence on how H19 and miR-17 contribute to the network of renal fibrosis remains unclear.

## Future Perspectives: LncRNA as a Novel Therapeutic Target for Kidney Disease

The activation of TGF-β/Smad signaling is one of the most characterized features in fibrosis. Although TGF-β is the crucial driver of fibrotic response, it also acts as an anti-inflammatory cytokine and essential mediator that regulates a wide range of biological processes in different cell types and disease conditions. Numerous studies reveal that lncRNAs participate in the emergence and progression of kidney diseases. An outline is becoming manifest in the contribution of TGF-β/Smad-mediated lncRNAs in renal fibrogenesis.

We are now getting better closer to understand how these lncRNAs regulate fibrosis. They can bind to the Smads proteins to exert either anti- or pro-fibrotic effects. They can also serve as miRNA sponges and interact with other signaling pathways to regulate ECM accumulation, EMT, MMT, or other fibrotic processes.

Based on the cell type-, tissue- and disease stage-dependent specialties, lncRNA may also present as biomarkers for clinical diagnosis in renal diseases (Brandenburger et al., 2018; Cheng et al., 2019; Li et al., 2019; Liu et al., 2019b; Loganathan et al., 2020). Interestingly, lncRNAs are relevant biomarkers for disease due to their existence with proteins or in vesicles in the extracellular space under pathological conditions (Teng and Ghoshal, 2015; Ellinger et al., 2016; Zhang et al., 2017c; E, S., Costa et al., 2018; Sarfi et al., 2019). Studies have demonstrated that circulating lncRNAs in body fluid, lncRNA GAS8-AS1, H19, metastasis-associated lung adenocarcinoma transcript 1 (MALAT1), and HOTAIR may be used as promising biomarkers to predict the early progression of cancers (Zhang et al., 2016, 2017b; Du et al., 2018a). Notably, the lncRNA expression profiles in urine also contribute to the early detection of acute T cell-mediated rejection of renal allografts (Lorenzen et al., 2015b), highlighting the importance of lncRNAs in T cell-mediated immune response during renal injuries (Hu et al., 2013).

The modulation of lncRNAs on renal fibrosis is a promising therapeutic target for fibrosis. However, it remains largely unexplored. The low expression amounts, the less conservation between species, the functional complexity, and the difficulty in modifying structures and locations of lncRNA in nuclear or cytoplasmic compartments have halted the development of lncRNA therapies.

Nevertheless, new technologies such as CRISPR/Cas9 editing (Wang et al., 2019; Horlbeck et al., 2020), Gapmer antisense oligonucleotide-mediated lncRNA silencing (Castanotto et al., 2015; Kuespert et al., 2020), plasmid/vector-delivery short hairpin RNAs (shRNAs) (Zhu et al., 2019; Yao et al., 2020) and ultrasound-mediated gene transfer method (Zhou et al., 2015a; Feng et al., 2018; Sun et al., 2018; Zhang et al., 2019c) may represent the novel strategies to modulate the expression and function of lncRNA in kidney diseases in the future.

## Author Contributions

Y-YG, J-YD, and X-RH wrote the manuscript, X-SL and H-YL revised and edited the manuscript. All authors contributed to the discussion of this manuscript.

## Conflict of Interest

The authors declare that the research was conducted in the absence of any commercial or financial relationships that could be construed as a potential conflict of interest.

## Footnotes

1 http://www.gencodegenes.org
